# Recent Advances in Structural Optimization of Quinazoline-Based Protein Kinase Inhibitors for Cancer Therapy (2021–Present)

**DOI:** 10.3390/molecules29040875

**Published:** 2024-02-16

**Authors:** Heba T. Abdel-Mohsen, Manal M. Anwar, Nesreen S. Ahmed, Somaia S. Abd El-Karim, Sameh H. Abdelwahed

**Affiliations:** 1 Chemistry of Natural and Microbial Products Department, Pharmaceutical and Drug Industries Research Institute, National Research Centre, El-Bohouth Street, Dokki, Cairo P.O. Box 12622, Egypt; ht.abdel-mohsen@nrc.sci.eg; 2 Department of Therapeutic Chemistry, Pharmaceutical and Drug Industries Research Institute, National Research Centre, El-Bohouth Street, Dokki, Cairo P.O. Box 12622, Egypt; mm.anwar@nrc.sci.eg (M.M.A.); ns.ismail@nrc.sci.eg (N.S.A.); ss.abdelkarim@nrc.sci.eg (S.S.A.E.-K.); 3 Department of Chemistry, Prairie View A & M University, Prairie View, TX 77446, USA

**Keywords:** quinazoline, design, synthesis, anticancer activity, structure–activity relationship, kinase inhibitors, molecular docking

## Abstract

Cancer is a complicated, multifaceted disease that can impact any organ in the body. Various chemotherapeutic agents have a low selectivity and are very toxic when used alone or in combination with others. Resistance is one of the most important hurdles that develop due to the use of many anticancer therapeutics. As a result, treating cancer requires a target-specific palliative care strategy. Remarkable scientific discoveries have shed light on several of the molecular mechanisms underlying cancer, resulting in the development of various targeted anticancer agents. One of the most important heterocyclic motifs is quinazoline, which has a wide range of biological uses and chemical reactivities. Newer, more sophisticated medications with quinazoline structures have been found in the last few years, and great strides have been made in creating effective protocols for building these pharmacologically active scaffolds. A new class of chemotherapeutic agents known as quinazoline-based derivatives possessing anticancer properties consists of several well-known compounds that block different protein kinases and other molecular targets. This review highlights recent updates (2021–2024) on various quinazoline-based derivatives acting against different protein kinases as anticancer chemotherapeutics. It also provides guidance for the design and synthesis of novel quinazoline analogues that could serve as lead compounds.

## 1. Introduction

Cancer is widely recognized as a serious public health issue and is universally acknowledged as the second-greatest cause of death in the United States [1]. According to the World Health Organization (WHO), the word “cancer” refers to a broad category of illnesses that can impact any area of the body [2]. Other terms used to characterize cancer are neoplasms and malignant tumors [3]. The fast growth of abnormal cells that eventually spread to other organs after initially residing in one area is one of the primary characteristics of cancer. This phenomenon is known as metastasis [4]. Widespread metastases are the main reason behind cancer-related deaths [4].

Recent WHO statistics indicate that 10 million deaths worldwide are expected to be related to cancer [5]. The International Agency for Research on Cancer (IARC) estimates that one in five people will develop cancer in their lifetime. One of the most important public health issues of the twenty-first century is cancer prevention [5]. Recent evidence suggests that effective primary prevention techniques could avoid at least 40% of cancer cases, and early tumor diagnosis could further reduce cancer-related death.

Protein kinases constitute one of the biggest protein families. By manipulating the location, activity, and functionality of many proteins via multisite phosphorylation, they regulate a broad spectrum of cellular processes [6]. Numerous critical cancer processes, such as tumor growth, metastasis, neovascularization, and chemotherapy resistance, have been shown to be significantly impacted by them. Protein kinases catalyze the transfer of a phosphate group from ATP to the hydroxy group of an amino acid residue. In cellular and molecular process, protein kinases are indispensable. As a result, they play a crucial part in the growth, dissemination, and survival of tumor cells in humans. Hence, this class of enzymes has drawn significant attention as a potential therapeutic target, with multiple kinase suppressors now receiving FDA approval for different cancer indications [7,8].

**Figure 1:**
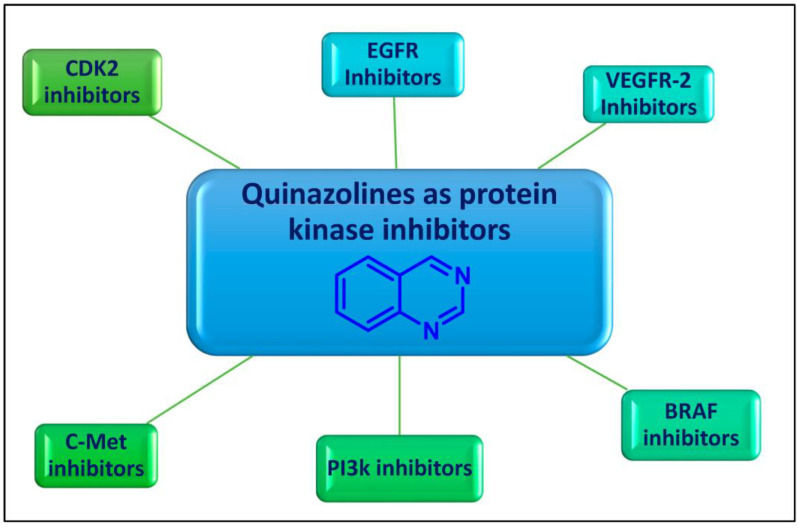
Protein kinase inhibitory activity of quinazolines for cancer therapy.

Quinazoline is an aromatic heterocyclic scaffold consisting of fused pyrimidine and a benzene ring. The quinazoline motif is considered to be one of the most important heterocyclic ring systems in pharmaceutical chemistry, present in many compounds and endowed with tremendous biological activities [9,10,11,12]. The quinazoline nucleus and its derivatives were reported to have significant potential as promising enzyme inhibitors (Figure 1).

## 2. Quinazolines as Protein Kinases Inhibitors

Over the years, diverse quinazolines have been discovered to act as single or multi-target protein kinase inhibitors. Epidermal growth factor receptor (EGFR), vascular endothelial growth factor receptor-2 (VEGFR-2), B rapidly accelerated fibrosarcoma (BRAF), phosphoinositide 3-kinase (PI3k), and mesenchymal–epithelial transition factor (c-Met) are among the protein kinases that are suppressed by quinazolines (Figure 1). FDA-approved medications based on quinazoline scaffolds and the latest developments in quinazolines as protein kinase suppressors for the treatment of cancer are the main topics of this review article.

### 2.1. Epidermal Growth Factor Receptor (EGFR) Inhibitors

One member of the ErbB family of the transmembrane tyrosine kinase receptors is the epidermal growth factor receptor (EGFR) [13]. It functions as a crucial mediator in cell proliferation, survival, adhesion, migration, and differentiation via downstream signaling transmission through auto-phosphorylation of several tyrosine residues following EGFR dimerization [13]. This receptor is activated when ligands, such as EGF or TGFa, bind to the extracellular domain, which sets off a signal transduction cascade that leads to cell proliferation, metastasis, and resistance to apoptosis in many types of cancers. Numerous malignancies, including those of the breast, ovary, colon, and non-small cell lung cancers (NSCLC), are linked to overexpression of EGFR [14].

A variety of quinazolines have been discovered in recent years to inhibit EGFR [15]. It is suggested that the 4-anilinoquinazoline scaffold interacts with ATP at the EGFR binding site [16]. Gefitinib (**1**) and erlotinib (**2**), the first-generation EGFR inhibitors, have received FDA approval for the treatment of non-small cell lung cancer (NSCLC) [17].

**Figure 2:**
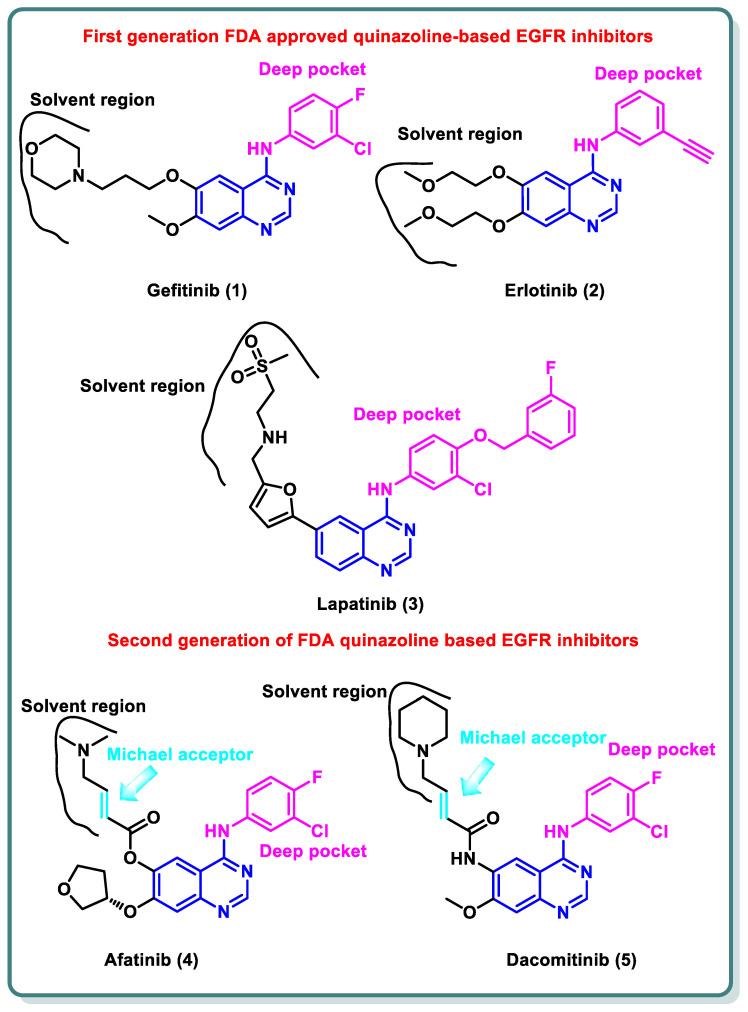
Structures of first- and second-generation FDA-approved quinazoline-based EGFR inhibitors.

Additionally, lapatinib (**3**) has received FDA approval for the management of certain cases of postmenopausal women’s breast cancer [18]. Despite the advancements made, many non-small cell lung cancers (NSCLCs) develop EGFR binding site mutations that promote tumor formation and carcinogenesis. The protein databank incorporates the crystal structure of EGFR co-crystalized with gefitinib (**1**), erlotinib (**2**), and lapatinib (**3**) [19,20]. These three inhibitors share the same binding mode to EGFR in which the quinazoline moiety occupies the ATP binding site and is stabilized by the ability of N1 to perform hydrogen bonding with the NH of Met793 in the hinge region, while N3 is involved in hydrogen bonding to Thr854 through a water molecule. The substituent at the 4-position is directed to the back of the ATP binding site and is involved in hydrophobic interactions with the protein, while the substitution at position 6 (and 7) is directed toward the solvent interface (Figure 2) [21].

Despite the advancements made, many NSCLCs develop EGFR binding site mutations that promote tumor formation and carcinogenesis. About 40% of NSCLC patients have an activating mutation of L858R, which raises the affinity for ATP and reduces the affinity for first-generation TKIs, resulting in resistance [22]. In contrast, about 50% of lung adenocarcinomas in patients who have become resistant to gefitinib (**1**) and erlotinib (**2**) exhibit the treatment resistance mutation T790M [23]. It entails changing threonine 790 at the gate region to a bulkier methionine, which prevents inhibitors from binding to the EGFR binding site. Hence, various structural alterations were made to the previous drugs to provide a range of ATP-competitive irreversible EGFR inhibitors that were recognized as second-generation, irreversible EGFR inhibitors [24]. Afatinib (**4**) and dacomitinib (**5**) occupy the ATP binding site of EGFR in the same manner as gefinitib (**1**); however, they are classified as irreversible EGFR inhibitors, as they have dimethylaminocrotonamide and 4-piperidin-1-yl-but-2-enamide, moieties, respectively, at the sixth position. These are capable of forming a covalent bond with the Cys797 present EGFR’s ATP binding site by acting as a Michael acceptor (Figure 2) [25].

**Figure 3:**
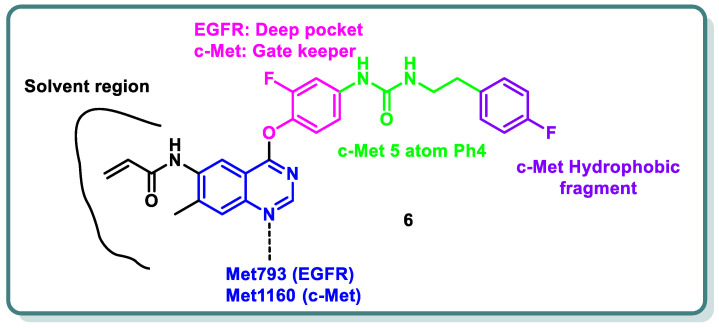
Design strategy of quinazoline derivative **6** as a dual EGFR and cMet inhibitor.

Additionally, different research groups have recently reported diverse scaffolds based on the quinazoline moiety as EGFR inhibitors beside other protein kinases. For example, in 2024, Han Zhang and his coworkers designed and synthesized novel 4-phenoxyquinazoline compounds as dual EGFR/c-Met suppressors to treat non-small cell lung cancer [26] (Figure 3). The most promising candidate is derivative **6**, which inhibits EGFR, EGFR_L858R/T790M_, and c-Met kinases at IC_50_ values of 64.8, 305.4, and 137.4 nM, respectively. It has significant anti-proliferative activity (IC_50_ = 2.27–3.35 µM), similar to that of afatinib, towards five types of cancer cell lines. Analysis of the cell cycle revealed that derivative **6** has the capacity to induce arrest at G2/M. In vivo findings in xenograft models demonstrated that derivative **6** might both cause apoptosis and suppress tumor development. Altogether, the results showed that derivative **6** is a novel compound with dual inhibitory activity against both EGFR and c-Met, and it can be considered a potential therapeutic medication to treat a variety of tumors (Figure 3) [26].

**Figure 4:**
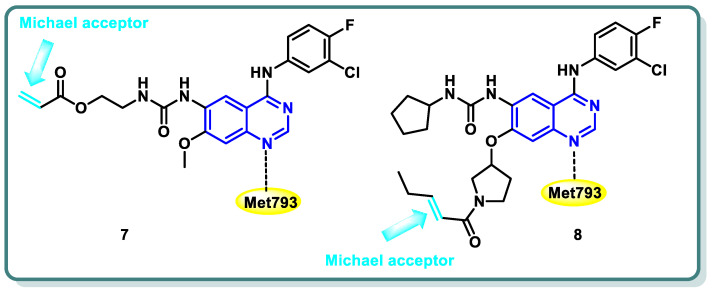
Structures of quinazoline derivatives **7** and **8**.

Diverse series of quinazolines were designed as EGFR_L858R/T790M_ inhibitors in 2022 by Wenhui Gan and coworkers [27]. Urea and thiourea moieties were incorporated in one series (compound **7** is a representative example, Figure 4), whereas a Michael receptor active warhead was introduced in another series (compound **8** is a representative example, Figure 4). Most of the designed and synthesized candidates demonstrated potent anti-proliferative impacts against different cell lines, including A549 and H1975 cancer cells. Additionally, they demonstrated moderate to outstanding kinase inhibitory efficacy against EGFR_WT_ and EGFR_L858R/T790M_. Urea derivative **7** revealed the most promising EGFR_WT_ and EGFR_L858R/T790M_ inhibitory activities (IC_50_ = 0.8 and 2.7 nM, respectively). Furthermore, both derivatives **7** and **8** strongly caused apoptosis of A549 cells. A cell cycle analysis showed that arrest of the cell cycle of A549 cells occurred at the S phase in derivative **7** and the G1 phase in derivative **8** [27] (Figure 4).

**Figure 5:**
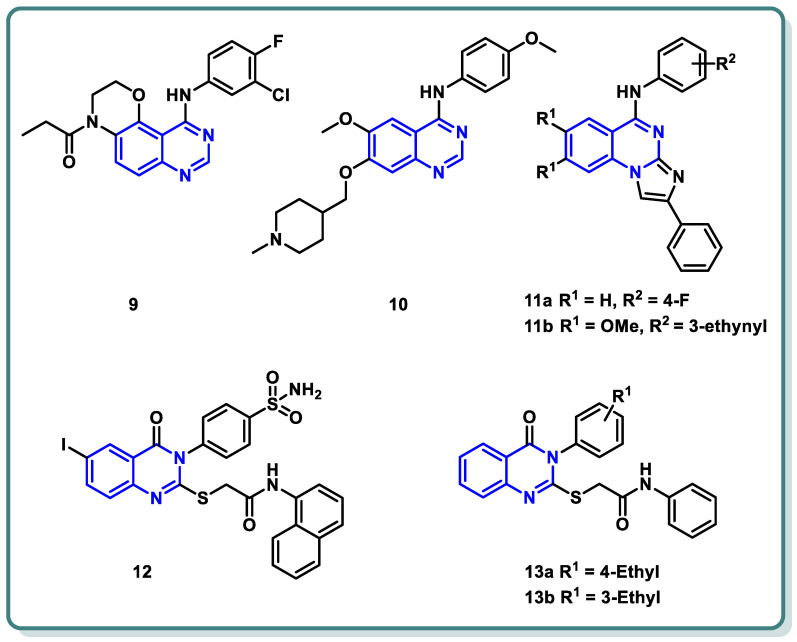
Chemical structures of various substituted quinazolines-based compounds **9**–**13** as EGFR inhibitors.

Moreover, a new family of 3,4-dihydro-2*H*-[1,4]oxazino [2,3-*f*]quinazoline derivatives that target EGFR was described by Qin et al. (Figure 5) [28]. With an IC_50_ ≤ 937.7 nM, the synthesized candidates showed strong EGFR inhibitory efficacy. Compound **9** (Figure 5) exhibited strong anti-proliferative properties against NCI-H1563 and H1975 cancer cell lines (IC_50_ = 25.69 nM). Furthermore, it produced an in vitro safety profile against the normal cell line, resembling 16HBE cells [28].

A series of 4-anilinoquinazoline analogues that are expected to be accommodated in the hinge region and allosteric pocket of EGFR_C797S_ was designed by Dou Dou [29]. When screening the designed and synthesized quinazolines regarding their inhibitory activity against EGFR_C797S_, derivative **10** (Figure 5) displayed a potent activity, with an IC_50_ = 0.128 µM. A potential anti-proliferative impact against BaF3-EGFR_L858R/T790M/C797S_ and BaF3-EGFR_19del/T790M/C797S_ was recorded for derivative **10** (IC_50_ = 0.75 and 0.09 μM, respectively). Additionally, derivative **10** demonstrated dose-dependent inhibition of EGFR and its downstream signaling cascades in BaF3-EGFR_19del/T790M/C797S_ cells. Interestingly, quinazoline **10** (Figure 5) demonstrated in vivo inhibition of tumor growth in a BaF3-EGFR_19del/T790M/C797S_ xenograft model (30 mg/kg, TGI = 67.95%) [29].

In 2023, a class of imidazo [1,2-*a*]quinazolines was presented by Hasenvand et al. as containing potent EGFR inhibitors [30]. When compared to erlotinib, the reference drug, imidazoquinazolines, **11a**,**b** (Figure 5) revealed significant anticancer activity against PC3, HepG2, HeLa, and MDA-MB-231, with IC_50_ values in the micro-molar range. Additional analyses showed that they could cause cell growth inhibition at the G0 phase of the cell cycle and induce apoptotic cell death. Two derivatives, **11a**,**b** were shown to have suppression activity and selectivity toward EGFR (as seen by their respective EGFR-IC_50_ = 82.0 µM and 12.3 µM, respectively). Furthermore, **11a**,**b** were shown via Western blot analysis to decrease extracellular signal-regulated kinase (ERK1/2) as well as EGFR phosphorylation. The degree of B-Actin phosphorylation, however, remained unchanged [30] (Figure 5).

In addition, Ghorab et al. introduced a series of novel quinazoline sulfonamide derivatives in 2023 [31]. Using the MTT assay, the compounds’ growth inhibitory activities against four cancer cell lines, namely HepG2, MCF-7, HCT116, and A549, were assessed. Based on the obtained findings, the compounds showing the most significant potencies were examined as EGFR_T790M_ and VEGFR-2 inhibitors. With an IC_50_ = 0.0977 µM against MCF-7, the quinazoline sulfonamide derivative **12** (Figure 5) exhibited the most noticeable cytotoxic effect and the most inhibitory activity against EGFR_T790M_ and VEGFR-2 (IC_50_ = 0.0728 and 0.0523 µM, respectively). In MCF-7 cells, compound **12** induced apoptosis and cell cycle stopping at the G2/M phase. Following an 8 Gy gamma irradiation dose, the radiosensitizing evaluation of compound **12** demonstrated its noteworthy capacity to sensitize the cancer cells to the impact of radiation. According to molecular docking simulation studies, compound **12** was predicted to occupy the VEGF and EGF receptors’ ATP-binding site and block their functions [31] (Figure 5).

The same research group designed and synthesized a class of *N*-substituted-2-((4-oxo-3-phenyl-3,4-dihydroquinazolin-2-yl)thio)acetamides [32]. Some of the synthesized candidates demonstrated potent cytotoxic activity on a HepG2 cell line with an IC_50_ reaching 1.11 µM. The quinazolines **13a** and **13b** inhibited EGFR (IC_50_ = 73.23 and 58.26 µM, respectively) in reference to erlotinib (IC_50_ = 9.79 µM) [32] (Figure 5).

### 2.2. Vascular Endothelia Growth Factor Receptor (VEGFR) Inhibitors

**Figure 6:**
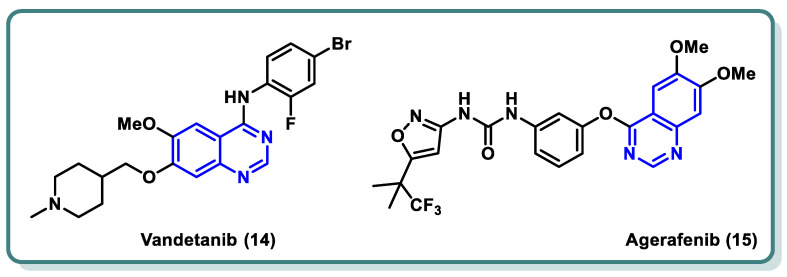
FDA-approved drugs as VEGFR-2 inhibitors for cancer treatment.

In both normal and pathological circumstances, the process of angiogenesis, or the formation of new blood vessels, is mediated by the tyrosine kinase system known as vascular endothelial growth factor (VEGF)/vascular endothelial growth factor receptor (VEGFR) [33,34,35]. When a solid tumor experiences hypoxia, massive levels of VEGF-A are released. By attaching itself to VEGFR-2, VEGF-A activates the receptor by starting a phosphorylation cascade. This then starts a signaling pathway downstream that eventually results in angiogenesis. When compared to healthy tissues, VEGFR-2 is the most expressed of the three VEGF receptors in a variety of cancer forms, including malignant melanoma, breast cancer, hepatocellular carcinoma, colon cancer, and so on [36,37,38]. Therefore, blocking VEGFR-2 is thought to be an effective way to prevent angiogenesis [38,39]. This ultimately leads to the cancer cells’ sources of nutrition and oxygen being blocked, which in turn prevents the cancer cells from surviving, proliferating, metastasizing, and dying off [40]. Vandetanib (**14**) is an example of a quinazoline derivative that acts as a VEGFR and EGFR inhibitor, and it has recently received a license from the FDA for the management of advanced medullary thyroid carcinoma [41,42]. Moreover, agerafenib (**15**) is a potential anti-tumor agent and potent mutlikinase (VEGFR-2, PDGFR, BRAF_WT_, and BRAF_V600E_) inhibitor [43] (Figure 6).

**Figure 7:**
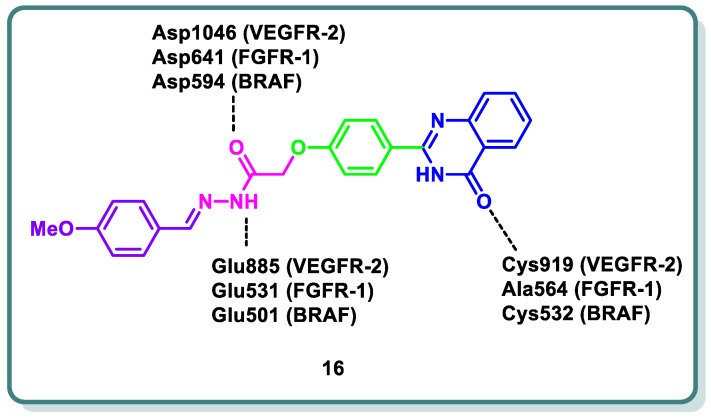
Design strategy of compound **16** as a VEGFR-2, FGFR-1, and BRAF inhibitor for cancer treatment.

Abd El-Karim et al. recently designed and discovered a novel class of quinazolinone *N*-acetohydrazides as significant inhibitors of VEGFR-2 alongside other kinases [44]. Compound **16** was the most potent candidate (Figure 7). It demonstrated IC_50_ values of 0.29, 0.35, 0.47, and 0.30 μM on VEGRF-2, FGFR-1, BRAF, and BRAF_V600E_, respectively. Also, compound **16** was the most potent candidate (Figure 7). It demonstrated IC_50_ values of 0.29, 0.35, 0.47, and 0.30 μM on VEGRF-2, FGFR-1, BRAF, and BRAF_V600E_, respectively. Additionally, compound **16** revealed growth inhibition potential against NCI-60 cancer cell lines (GI_50_ = 1.64 μM). In addition, the same study showed that compound **16** led to cell cycle arrest in the MDA-MB-231 cell line at the G2/M phase alongside an apoptotic effect, as observed based on the elevation in the percentage of cells in the early and late apoptosis phases from 0.05 and 0.99% in control cells to 0.44% and 2.43%, respectively. The newly quinazolines exhibit analogous binding mechanisms to the protein kinase active sites under study, as demonstrated by in silico molecular docking simulations. This mechanism represents the settlement of the quinazolinone scaffold within the hinge region of the target kinases, where the C=O forms a hydrogen bond with the important amino acids. Meanwhile, the essential amino acids in the gate area interact through hydrogen bonding with the *N*-acetohydrazide linker and the peripheral phenyl moiety occupies the allosteric hydrophobic back pocket, producing a number of hydrophobic interactions (Figure 7) [44].

**Figure 8:**
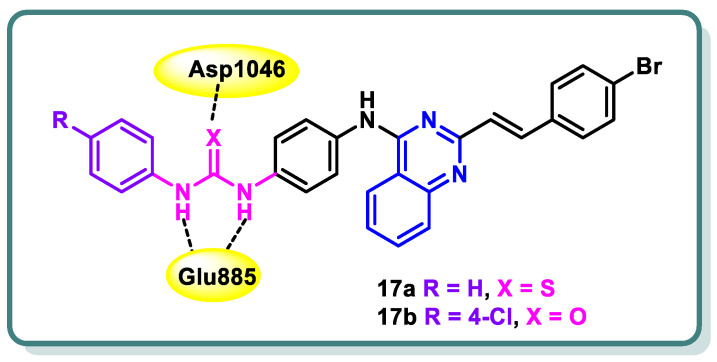
Design of quinazolines **17a** and **17b** as VEGFR-2 inhibitors.

Hamdi and colleagues [45] have successfully synthesized a novel series of 4-anilino-2-vinylquinazolines. The evaluation encompassed the assessment of in vitro anticancer activity, suppression of VEGFR-2, and activation of apoptosis. Among the products that underwent testing, compounds **17a** and **17b** (Figure 8) demonstrated significant potencies as anticancer agents against HCT-116, HepG-2, and MCF-7 cancer cell lines. Their effectiveness, with IC_50_ values ranging from approximately 4.92 to 11.31 μM, was found to be equivalent to that of sorafenib, which exhibited IC_50_ values ranging from approximately 5.47 to 9.18 μM. In addition, compound **17a** demonstrated a significant suppression impact against VEGFR-2, with an IC_50_ value of 60.27 nM, which is similar to the inhibitory activity of sorafenib (IC_50_ = 55.43 nM). On the other hand, compound **17b** showed a moderate suppression effect against VEGFR-2 (IC_50_ = 93.5 nM). Compound **17a** exhibited a notable level of apoptosis-inducing activity, amounting to 36.24%, when tested on MCF-7 cancer cells. This efficacy is equivalent to the apoptotic impact demonstrated by sorafenib, which amounted to 32.46%. Furthermore, compound **17a** was found to stop the cell cycle at the G1/S phase. The anticancer and VEGFR-2 inhibitory effects were elucidated using molecular modeling investigations, which demonstrated that compound **17a** possesses the crucial element necessary for interacting with the DFG-binding domain, a critical factor in VEGFR-2 inhibition. Furthermore, compound **17a** exhibited molecular fields that were comparable to those observed by sorafenib (Figure 8) [45].

**Figure 9:**
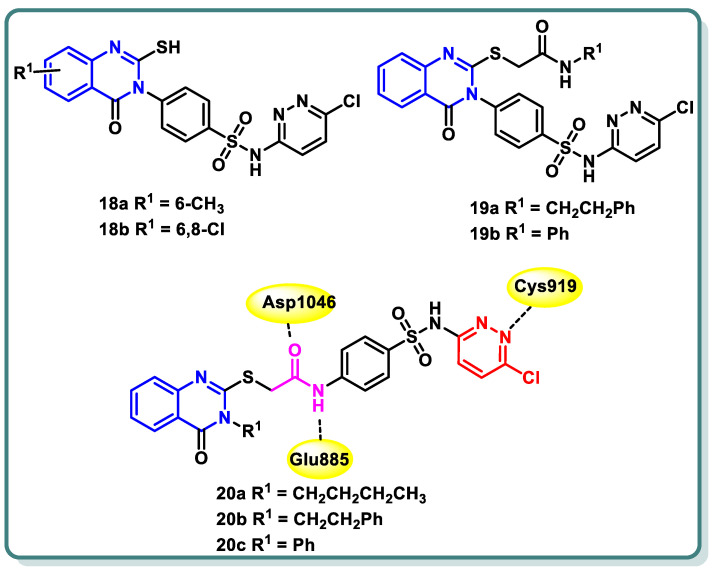
Structures of sulfachloropyridazine-bearing quinazoline-4(3*H*)-one derivatives **18a**,**b**, **19a**,**b** and **20a**–**c**.

In their study, Zahran et al. [46] documented the development and creation of innovative sulfachloropyridazine-bearing quinazoline-4(3*H*)-one derivatives. The cytotoxic activity of all the produced compounds was evaluated in vitro at a dose of 10 μM against a panel of 60 human cancer cell lines. Compounds **18a**, **18b**, **19a**, **19b**, **20a**, **20b**, and **20c** (Figure 9), which exhibited the highest levels of activity, were chosen for the purpose of assessing their capacity to suppress VEGFR-2 in comparison with sorafenib and vandetanib as standard medicines. Thioacetamide derivative **20b** exhibited the highest level of inhibitory action against VEGFR-2, with an IC_50_ value of 66 nM. Additionally, the assessment of the antiangiogenic characteristics necessary for inhibiting wound healing in HUVEC cells was carried out for **20b**, which exhibited a closure percentage of 58.52%, which is similar to that of sorafenib (46.67%). The analogue **20b** exhibited moderate suppression activity against PDGFR (IC_50_ = 180 ± 0.009 nM), EGFR (IC_50_ = 98 ± 0.004 nM), and FGFR-1 (IC_50_ = 82 ± 0.004 nM) as well. The Annexin V-FITC/PI test showed that **20b** notably enhanced early apoptosis. Additionally, it led to an elevation in the levels of active caspase-3 and Bax while simultaneously decreasing the levels of Bcl-2 in UO-31 cells compared to the untreated control group. The molecular docking analysis revealed that the highly active compounds exhibited a favorable binding conformation inside the active site of VEGFR-2, suggesting their potential role in exerting anticancer effects (Figure 9) [46].

**Figure 10:**
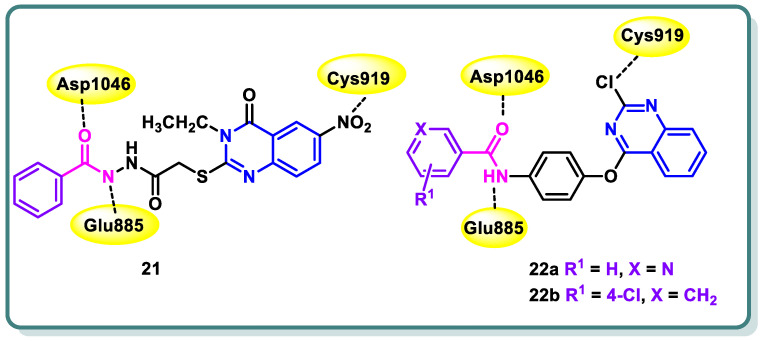
Quinazolines **21** and **22** showing their expected interactions with the VEGFR-2 active site.

Additionally, in 2021, Abdallah and coworkers [47] reported the design, synthesis, and biological assessment of nineteen derivatives of quinazolin-4-one. These compounds were investigated for their potential anticancer action, taking into consideration the pharmacophoric properties of VEGFR-2 kinase inhibitors. The thioacetyl-benzohydrazide compound **21** exhibited the highest potency among the tested candidates. It demonstrated an IC_50_ value of 4.6 ± 0.06 μM against VEGFR-2 kinase. Furthermore, it demonstrated IC_50_ values of 17.23 ± 1.5, 26.10 ± 2.2, and 30.85 ± 2.3 μg/mL against HepG2, PC3, and MCF cell lines, respectively. Simultaneously, the experiment yielded an IC_50_ value of 145.93 ± 1.1 μg/mL when tested against the normal human lung fibroblasts cell line (WI-38), suggesting a favorable selectivity index. Additional examination of the HepG2 cell cycle revealed that compound **21** possesses the capacity to trigger apoptosis and halt cell proliferation specifically at the G2/M stage (Figure 10). In addition, the docking investigations revealed that compound **21** possesses the capability to effectively connect to VEGFR-2 by forming three crucial hydrogen bonds with the critical residues Glu885, Asp1046, and Cys919, as depicted in Figure 10. In conclusion, the study proposes that compound **21** exhibits potential as a primary candidate for the advancement of efficacious anticancer medicines that specifically target VEGFR-2 [47].

One year later, the same research group synthesized **17** novel compounds by utilizing the quinazoline, quinoxaline, and benzene nuclei [48]. The strategy of their work was to align with the known pharmacophoric characteristics of type II VEGFR-2 inhibitors. The biological findings indicated that the quinazoline derivatives **22a** and **22b** (Figure 10) exhibited notable potential as anticancer medicines against the hepatocellular carcinoma cell line (HepG2) that specifically target VEGFR-2 kinase. In the VEGFR-2 inhibitory experiment, compounds **22a** and **22b** displayed IC_50_ values of 60.00 and 86.36 nM, respectively, whereas sorafenib, the positive control, displayed an IC_50_ value of 54.00 nM. Simultaneously, the IC_50_ values acquired for the two contenders against HepG2 were 24.10 and 17.39 mM, respectively. Upon further investigation, compound **22a** was found to possess significant potential as an inducer of apoptosis in HepG2 cells. Compound **22a** increased the rate of apoptosis from 1.20% to 12.46% by upregulating the expression levels of caspase-3, BAX, and P53 and reducing the level of Bcl-2 (Figure 10) [48].

**Figure 11:**
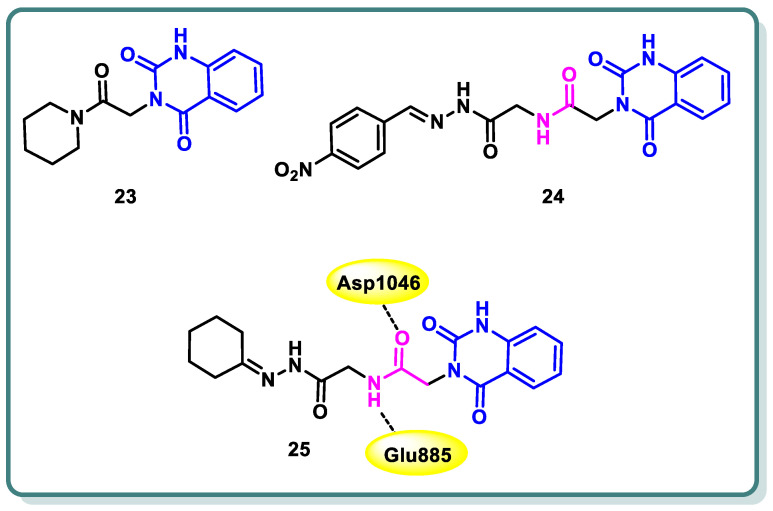
Structures of quinazolines **23**, **24**, and **25**.

In a study conducted by Hassan et al. [49], the authors developed a series of 3-substituted quinazoline-2,4(1*H*,3*H*)-diones using novel synthetic methodologies with the aim of creating dual inhibitors of c-Met and VEGFR-2 tyrosine kinases. The antiproliferative efficacy of the target compounds was evaluated using the HCT-116 colon cancer cell line, which overexpresses c-Met/VEGFR-2. Among the tested compounds **23**, **24**, and **25** (Figure 11), a significant inhibition was observed for both c-Met and VEGFR-2 tyrosine kinases, with IC_50_ values ranging from 0.052 to 0.084 µM. The molecular docking study revealed that compound **25** displayed hydrogen bonding with Glu885 and Asp1046 (Figure 11) [49].

**Figure 12:**
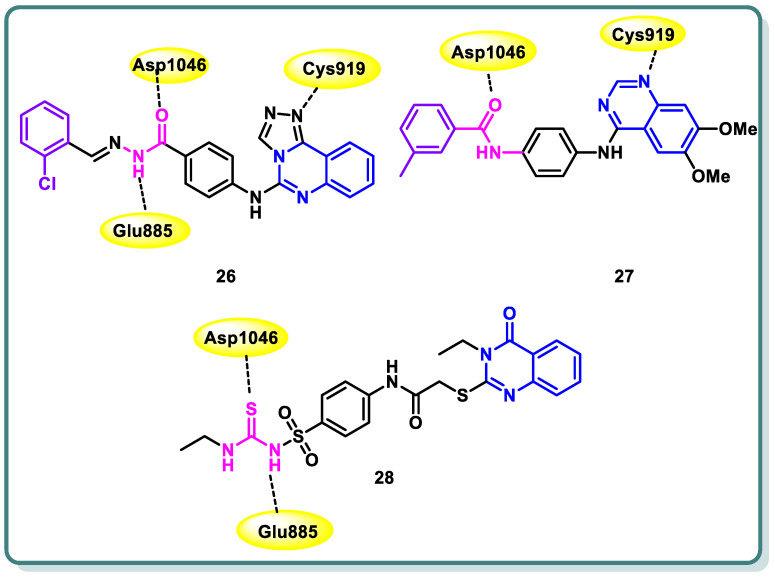
Structures of quinazoline derivatives **26–28**.

Furthermore, Azab and colleagues [50] designed and synthesized new compounds belonging to the [1,2,4]triazolo [4,3-*c*]quinazoline class. These compounds were strategically intended to possess the crucial pharmacophoric characteristics necessary for inhibiting VEGFR-2. The aim of this study was to assess the antiproliferative impacts of the synthesized compounds on two tumor cell lines, namely HepG2 and HCT-116. Sorafenib was employed as a positive control in this investigation. Compound **26** demonstrated significant potential antiproliferative activity (IC_50_ = 4.88 and 5.21 μM, respectively). Furthermore, the results demonstrated that it had the most potent inhibitory effect against VEGFR-2, as indicated by an IC_50_ value of 53.81 nM, surpassing that of sorafenib (IC_50_ = 44.34 nM). The cell cycle analysis’s findings showed that compound **26** can cause cell cycle stoppage in HepG2 cells, impacting both the S and G2/M stages. Furthermore, the administration of this substance resulted in a tenfold augmentation in the number of apoptotic cells as compared to the control group. In addition, the administration of this chemical resulted in a significant 3.35-fold upregulation of BAX expression levels, accompanied by a modest 1.25-fold down regulation of Bcl-2 expression levels. The ratio of BAX to Bcl-2 was determined to be 4.57, suggesting a favorable apoptotic outcome. Furthermore, the results demonstrated a notable elevation in the expression of caspase-3 (4.12- fold) in comparison to the control cells (Figure 12) [50].

Wang et al. reported the design and synthesis of new 6,7-dimethoxy-4-anilinoquinazoline derivatives and assessed them as potent VEGFR-2 inhibitors [51]. Among the target derivatives, methylbenzamide compound **27** showed the most significant VEGFR-2 kinase inhibitory activity (IC_50_ = 0.016 ± 0.002 μM), better than sorafenib (IC_50_ = 0.021 ± 0.004 μM). Furthermore, it showed the highest antiproliferative effect against Hep-G2 and MCF-7, with IC_50_ values in the low-micromolar range (Figure 12). In addition, the target compound was docked into the ATP binding site of VEGFR-2 (PDB: 3B8Q) using the Discovery Studio 4.5/CDOCKER protocol. Figure 12 illustrates the interaction mechanism between **14b** and VEGFR-2. It demonstrates that the incorporation into the ATP-binding site of VEGFR-2 was through various interactions, including hydrogen bonds, π–cation interactions, π–anion interactions, π–π stacked interactions, alkyl interactions, and van der Waals interactions with the amino acid residues of VEGFR-2.

A novel set of quinazolin-4 (3*H*)-ones was also conceptualized, synthesized with the intention of functioning as inhibitors of VEGFR-2 [52], and subsequently assessed for their efficacy in impeding the proliferation of hepatocellular carcinoma, specifically targeting the HepG-2 cell line. Compound **28** exhibited greater potency compared to doxorubicin and 78% of the sorafenib activity. Compound **28** displayed an IC_50_ value of 4.33 ± 0.2 μg/mL, while doxorubicin showed an IC_50_ value of 4.50 ± 0.2 μg/mL, and sorafenib exhibited an IC_50_ value of 3.40 ± 0.25 μg/mL. In addition, compound **28** significantly inhibited VEGFR-2, with IC_50_ values of 3.1 ± 0.04 μM compared to the IC_50_ value of sorafenib, which was found to be 2.4 ± 0.05 μM. Moreover, compound **28** was found to cause apoptosis and halt the progression of the cell cycle at the G2/M phase. Furthermore, the in vivo anticancer studies demonstrated that compounds **28** exhibited a substantial reduction in tumor growth. The immuno-histochemical analysis of activated caspase-3 expression indicated a progressive rise in the levels of cleaved caspase-3 protein over time following the administration of compound **28** to HepG-2 cells. In addition, the fibroblastic proliferative index test demonstrated that compound **28** exhibited the ability to mitigate liver fibrosis. The findings obtained from the biological screening were further validated through docking studies (Figure 12) [52].

**Figure 13:**
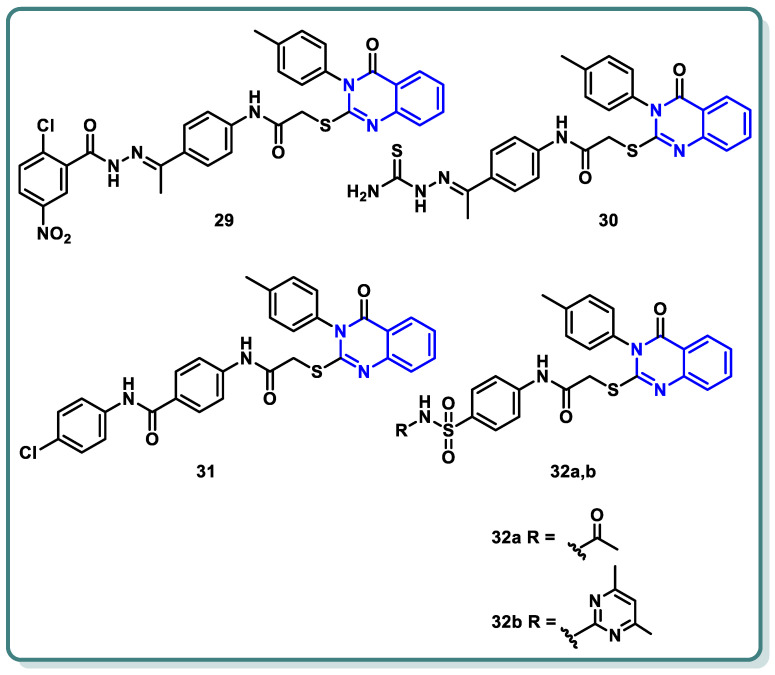
Structures of quinazolines **29**–**32**.

A new series of different quinazoline derivatives of potential cytotoxic and VEGFR-2 inhibitory activities were designed and synthesized by El-Adl et al. [53]. Compounds **29**, **30**, **31**, **32a**, and **32b** produced significant impacts. They were found to be mostly more active than the standard or almost the same, with IC_50_ results varying between 1.68 ± 0.06 and 9.89 ± 0.39 μM compared to doxorubicin of IC_50_ values, which varied from 8.28 ± 0.33 to 9.63 ± 0.38 μM, and with sorafenib of IC_50_ values ranging from 7.31 ± 0.29 to 9.40 ± 0.37 μM against HepG2, HCT-116, and MCF-7 cells, respectively. An in vitro VEGFR-2 enzyme assay showed that the latter five compounds, **29**, **30**, **31**, **32a**, and **32b** significantly suppressed VEGFR-2 (IC_50_ = 0.290 ± 0.05, 0.517 ± 0.07, 0.380 ± 0.04, 0.377 ± 0.04, and 0.415 ± 0.03 μg/mL, respectively) compared to sorafenib, with an IC_50_ value of 0.588 μg/mL (Figure 13) [53].

**Figure 14:**
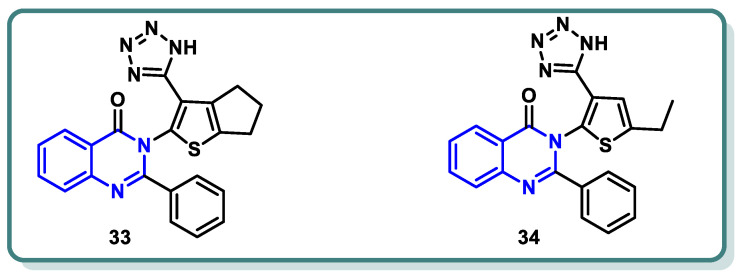
Structures of VEGFR-2 inhibitors **33** and **34**.

In a recent study conducted by Elrayess et al. [54], novel derivatives of 2-phenylquinazolin-4(3*H*)-one were developed. Their cytotoxic effects were assessed in situ against MCF-7 and HepG2 cells, revealing promising results. Derivatives **33** and **34** (Figure 14) exhibited notable antiproliferative effects against MCF-7 (IC_50_ = 1.35 μM) and HepG2 cells (IC_50_ = 3.24 μM), which were equivalent to those of sorafenib (IC_50_ values of 3.04 and 2.93 μM, respectively). When comparing compounds **33** and **34** to sorafenib as VEGFR-2 inhibitors, they exhibited better activities, with IC_50_ values of 13, 67, and 30 nM, respectively. Compound **33** boosted cell cycle arrest at the S-phase by 55.11 times by inducing apoptosis in breast cancer cells. This compound downregulated the antiapoptotic gene Bcl-2 and elevated the apoptotic-related genes caspases 3, 8, 9, P53, Bax, and others. Molecular docking studies revealed that compound **33** exhibited a considerable binding affinity to both the DFG loop and the allosteric region of VEGFR-2 (Figure 14).

### 2.3. Rapidly Accelerated Fibrosarcoma (RAF) Inhibitors

The three isoforms of rapidly accelerated fibrosarcoma (RAF) kinases, CRAF (RAF-1), BRAF, and ARAF, are serine/threonine protein kinases that are a component of the MAPK signaling cascade, a critical promoter involved in cellular development, differentiation, and proliferation [55]. Compared to ARAF and CRAF, BRAF is the most significant RAF kinase and an essential MEK1/2 activator [55]. Numerous forms of melanoma are commonly linked to BRAF mutations [41]. The most prevalent BRAF mutation is known as BRAF (V600E), in which a glutamate residue is placed in place of a valine residue at position 600 [41]. CRAF is activated via interactions with BRAF in a RAS-dependent process, which represents the complexity of the signaling pathway. The quinazolinone nucleus is a privileged scaffold with various derivatives acting as RAF inhibitors. While GNE-0749 (**36**), a fluoroquinazolinone urea derivative, acts as a highly selective pan-RAF suppressor in K-Ras mutant animals in pre-clinical research [56], AZ628 (**35**) is a quinazolinone derivative that acts as a BRAF (wt), BRAF (V600E), CRAF, and VEGFR-2 multi-kinase inhibitor [57].

**Figure 15:**
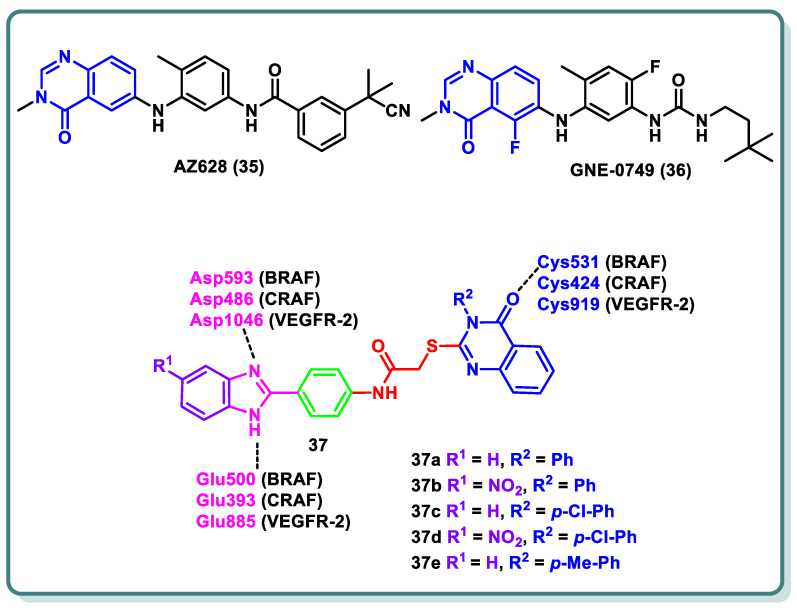
Structures of various quinazolines as RAF-inhibiting candidates.

In 2022, Ali and coauthors designed and synthesized of a series of 2-arylbenzimidazole-thioquinazolin-4(3*H*)-ones conjugates **37** (Figure 15) [58]. Dual potency on BRAF_V600E_ and CRAF was found for **37a**–**d** (IC_50_ values 0.002–0.37 µM). Additionally, multi-kinase inhibitory activity was observed for the quinazolinone derivative **37e** (IC_50_ = 6.10, 6.70, 2.50, 10.80, 0.03, 0.13, and 0.12 µM a on VEGFR-2, BRAF_WT_, BRAF_V600E_, CRAF, PDGFR-β, FLT-3, and c-KIT, respectively). The synthesized compounds’ growth inhibition percentage (GI%) was determined at 10 µM after they were assessed in vitro against a panel of anticancer cell lines at NCI–US. Among the compounds chosen for screening in the five-dose trial, compounds **37c**,**d** exhibited GI50 values as high as 1.1 µM. Moreover, a cell cycle analysis was carried out in an A-375 melanoma cell line for compound **37c**, which arrested the cell cycle at the G2/M phase by 7.89%, as well as induction of the late apoptosis phase by 4.14%, respectively. Molecular docking experiments were conducted on BRAF, CRAF, and VEGFR-2 to further investigate the binding mechanisms for the compounds and their interactions via the important amino acids, namely BRAF (Glu500, Cys531, and Asp593), CRAF (Glu393, Cys424, and Asp486), and VEGFR-2 (Glu885, Cys919, and Asp1046) (Figure 15) [58].

### 2.4. Phosphoinositide 3-Kinase (PI3K) Inhibitors

Phosphoinositide 3-kinase, or PI3K, is an intracellular lipid kinase that is involved in the regulation of cell proliferation, migration, differentiation, and death. It is part of the PI3K/AKT/mTOR signaling pathway [59].

**Figure 16:**
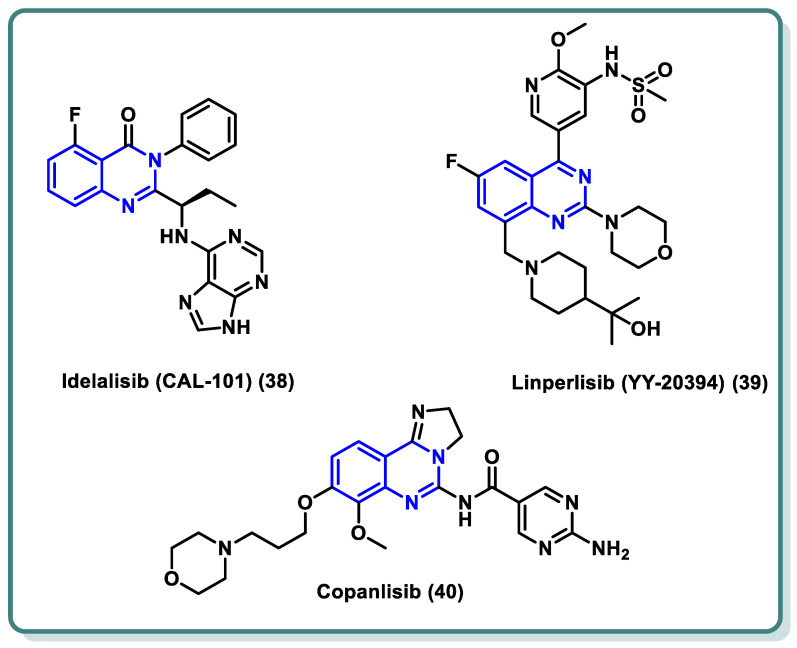
Structures of different PI3K inhibitors **38**–**40** based on the quinazoline nucleus.

PI3Ks can be divided into three categories based on their structural characteristics and substrate selectivity, with class I PI3K receiving the most research attention. G protein-coupled receptor (GPCR) or receptor tyrosine kinase (RTK) signaling is mediated by class I PI3K [60,61]. The active class I PI3K is catalyzed by signals from the RTK and GPCR to phosphorylate PIP2 to PIP3. This lipid product participates in the phosphorylation of protein kinase B (PKB, Akt) and functions as a second messenger to regulate cellular processes. Class I PI3K is further classified into PI3Kα, PI3Kβ, PI3Kδ, and PI3Kγ [62]. Research has demonstrated a strong correlation between PI3K dysregulation and the emergence and progression of a wide range of tumors [63]. Specifically, PI3Kδ is a significant subtype of type I PI3K that primarily regulates B cell receptor (BCR) signaling [64]. As a result, PI3Kδ is now a useful target for the treatment of B cell-related illnesses as well as hematologic malignancies. In recent years, a number of quinazoline-based PI3Kδ inhibitors have been approved by the FDA in the past decade for the treatment of malignant tumors, asthma, immunodeficiency, and autoimmune diseases. As an illustration, idelalisib (**38**) (Figure 16), the first PI3Kδ inhibitor licensed for therapeutic use, was used to treat relapsed follicular lymphoma (FL), small lymphocytic lymphoma (SLL), and chronic lymphocytic leukaemia (CLL) [65]. Idelalisib occupies the ATP binding site of PI3Kδ, where the purine moiety is involved in hydrogen bonding through N3 and N9 with the amino acid residues Val-828 and Glu-826, respectively. Additionally, N7 of the purine moiety forms a water-mediated network with Asp911 in conjunction with N1 of the quinazoline moiety. Moreover, the inhibitor showed additional hydrophobic interaction through its ethyl group with the target protein [66]. Furthermore, linperlisib (**39**) (Figure 16) was successively approved for treating CLL, SLL, FL, and APDS (activated PI3Kδ syndrome) [67]. Also, Copanlisib (**40**) has been indicated for various cancers [68,69].

**Figure 17:**
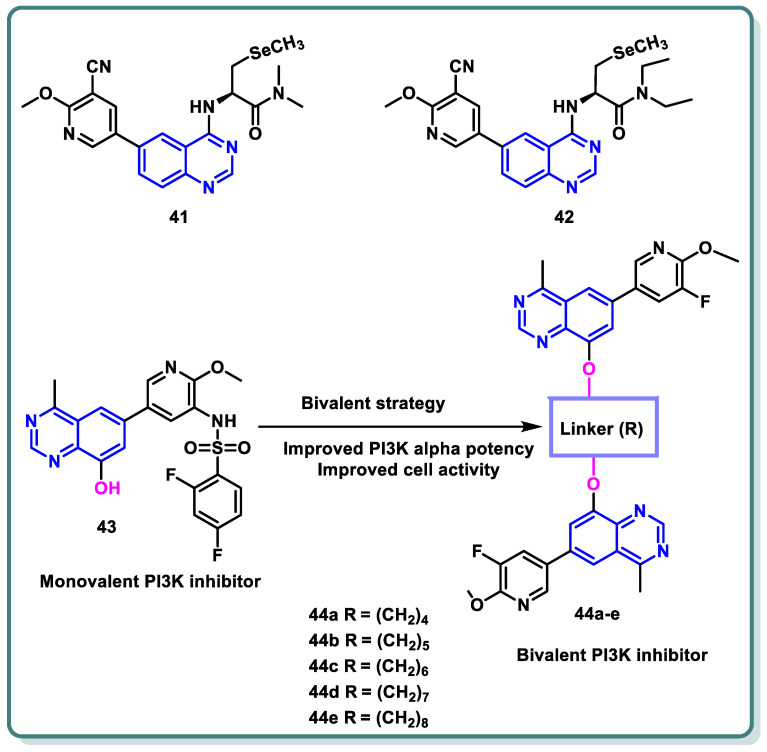
Structure of different quinazoline-based derivatives as PI3K inhibitors.

In a recent study by Gao et al., a series of selenium-containing PI3Kδ inhibitors based on quinazoline and pyrido [3,2-*d*]pyrimidine skeletons was designed and synthesized. Following the assessment of δ-kinase activities, the obtained data demonstrated that **41** and **42** tailed with a line-chain amine produced potent suppression activity against PI3Kδ (IC_50_ = 1.13 and 2.52 nM, respectively) [70]. Ab in vitro anticancer assay against the human malignant B cell line SU-DHL-6 demonstrated the promising activity of these compounds compared with idelalisib as a positive control (Figure 17).

Furthermore, in order to create a number of bivalent PI3K inhibitors, Xia and colleagues selected a 4-methylquinazoline scaffold (Figure 17). To be more precise, legend **43** was chosen as the monovalent counterpart that might generate three important interactions with the PI3Kα target: a charged interaction between Lys802 and the deprotonated sulfonamide, a water bridge via pyridine nitrogen and Tyr836 and Asp810, and a hydrogen bond between the nitrogen atom in the quinazoline scaffold at position 3 and Val851. The quinazoline scaffold’s position 8 was appropriate for a range of functional groups that occupied the solvent-exposed region [71]. As a result, several bivalent PI3K inhibitors were created by adding linkers of various kinds and lengths to the quinazoline scaffold’s 8 locations (Figure 17), keeping the major structural groups attached to PI3Kα’s ATP-binding pocket. A variety of scaffold substituents (R) were investigated to identify the ideal “monovalent counterpart.” By blocking the PI3K pathway, these bivalent PI3K inhibitors are anticipated to exhibit greater PI3K potencies and offer a novel approach to the search for new cancer treatments. Different lengths of alkylene linkers were bonded as hydrophobic segments to the phenolic group in position 8 of the 4-methyl quinazoline scaffold using ether bonds. It was demonstrated that compound **44a**, bearing a 4- methylene linker suppressed PI3K*α* (IC_50_ value of 1166 nM), was roughly 2-fold more significant than compound **44b**, bearing a 5-methylene linker (IC_50_ = 2722 nM). Compound **44c**, bearing 6-methylene linker, showed a 2-fold potency more than that of compound **44b** (IC_50_ = 1282 nM). In addition, compounds **44d** and **44e**, which have 7- and 8-methylene linkers, exhibited lower PI3K potencies than compound **44c**. Their IC_50_ values were 2137 nM and 4247 nM, respectively. With an additional chain length extension from the 9-methylene linker to 12-methylene linker, the inhibitory effects on PI3Kα appeared to be diminished (IC_50_ > 10,000 nM).

### 2.5. Cyclin-Dependent Kinase (CDK) Inhibitors

Cyclin-dependent kinases (CDKs) are a family of protein kinases with central roles in orchestrating the eukaryotic cell cycle. Among the 13 mammalian CDKs, CDK1, CDK2, CDK3, CDK4, and CDK6 hold particular significance for their control of cell cycle progression through targeted phosphorylation of protein substrates [72]. Dysregulation of these kinases, leading to uncontrolled cell proliferation, is a hallmark of cancer [73]. CDK2 [74,75,76,77,78,79] occupies a pivotal position in this regulatory network. Its influence extends beyond cell cycle transitions and G1/S and G2 phase progression to encompass DNA repair [80], gene transcription, and epigenetic modifications in tumors [81,82,83]. Abnormal expression of cyclins, the regulatory subunits essential for CDK2 activity, further underscores its oncogenic potential, particularly through overexpression of cyclin A and/or E in various cancers [84,85].

Recognizing CDK2’s multifaceted role in cancer development, researchers have focused on its inhibition as a therapeutic strategy. Potent CDK2 inhibitors, such as dinaciclib [86], milciclib [80], and roniciclib [87], are currently undergoing clinical evaluation, targeting this kinase specifically or in combination with others. However, the presence of adverse effects observed in clinical trials underscores the need for further refinement and discovery of more selective and efficacious CDK2 inhibitors [88].

**Figure 18:**
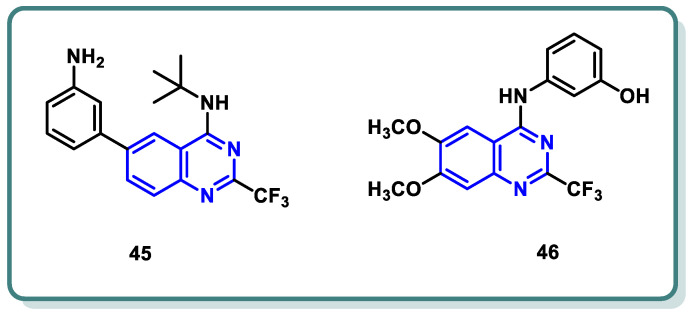
Reported quinazoline-based CDK2 inhibitors.

Our analysis of the literature revealed a diverse landscape of CDK2 inhibitors, encompassing scaffolds like pyrazolopyrimidines (dinaciclib), purines (fadraciclib [89], roscovitine [90]), and quinazolines (compounds **45** [91] and **46** [92]) (Figure 18).

**Figure 19:**
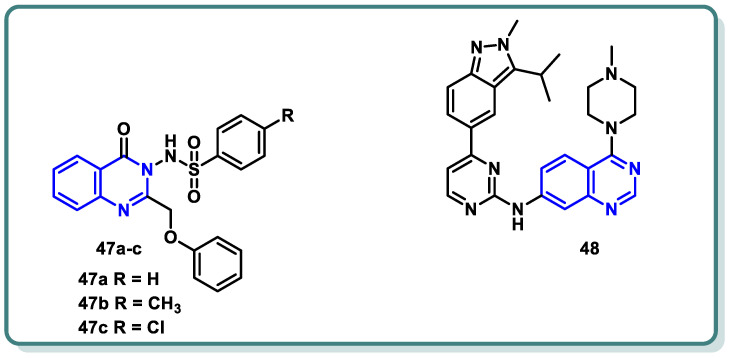
Quinazolines **47** and **48** as CDK inhibitors.

Recently, Mohammed et al. [93] strategically chose a substitution pattern at the 2 and 3 positions of the quinazolinone ring to create distinct electronic circumstances (Figure 19). This variation was intended to impact the lipophilicity, thereby influencing the activity of the target molecules. The objective behind the synthesis these analogues were to develop active CDK2 suppressors with enhanced efficacy against cancer cells characterized by overexpression of CDK2. In this study, quinazolin-4(3*H*)-one derivatives **47** were synthesized as CDK2 inhibitors, showing potent antiproliferative activity against melanoma (MDA-MB-435) and glioblastoma (SNB-75) cell lines. Compound **47c** demonstrated a superior potency, with an IC_50_ of 3.03 µM against MDA-MB-435, surpassing the reference roscovitine [90]. Additionally, **47c** induced cell cycle arrest and apoptosis, highlighting CDK2 as a potential target. CDK2 inhibitory assays confirmed the potency of **47c** (IC_50_ = 0.63 µM). Additionally, computational ADME studies indicated that the most active compound **47c** possessed promising pharmacokinetic properties. These findings endorse the potential of compound **47c** as a valuable lead candidate in the quest for promising anti-melanoma agents through CDK2 inhibition.

On the other hand, investigations by Huang et al. [94] revealed that compound **48** exhibits robust multi-targeted inhibition of cyclin-dependent kinases (CDKs), encompassing CDK2, CDK1, CDK4, CDK8, and CDK9. These results led to G1 arrest and pro-apoptotic signaling in hematologic malignancies. The inclusive approach of compound **48** addressed the potential resistance mechanisms, broadening its impact across a spectrum of vulnerable cancer cells. While compound **48** showed promise in preclinical studies, further research is essential to fully unravelling the potential of compound **48**, paving the way for transformative clinical trials in the complex landscape of hematologic cancers.

## 3. Conclusions

Research on new cancer treatments has progressed from a time of harmful, non-selective medications to safer, selective ones. The main shortcomings of chemotherapeutic drugs are their lack of selectivity and the toxicity that follows. Accordingly, there is significant evidence to support the improvement of targeted therapy with site-specific action in the management of this fatal illness. Extensive work in the drug discovery field and understanding of cancer biology led to massive exploration of quinazoline pharmacophore-based derivatives as a kinase-targeted cancer therapy. Several quinazoline-based drugs acting as kinase inhibitors have been licensed by the FDA and are available for clinical use for the treatment of advanced cancer diseases. The majority of the alterations that were made to the quinazoline skeleton at positions 4, 6, and 7 in order to create anticancer molecules produced substances with extremely strong biological activities against cancer. Additional structural alterations to the quinazoline nucleus may result in novel derivatives with a greater efficacy than already prescribed medications. The literature review above highlights the significance of quinazoline nucleus structural modifications and resulting molecular changes for cancer therapy. This study provides scientists working towards improvements in novel target-specific therapeutic candidates for cancer treatment with fresh insights and an enhanced comprehension.

## Data Availability

The authors confirm that the data supporting the findings of this study are available within the article references.
